# Identifying Risk: Concurrent Overlap of the Antarctic Krill Fishery with Krill-Dependent Predators in the Scotia Sea

**DOI:** 10.1371/journal.pone.0170132

**Published:** 2017-01-13

**Authors:** Jefferson T. Hinke, Anthony M. Cossio, Michael E. Goebel, Christian S. Reiss, Wayne Z. Trivelpiece, George M. Watters

**Affiliations:** Antarctic Ecosystem Research Division, Southwest Fisheries Science Center, National Marine Fisheries Service, National Oceanic and Atmospheric Administration, La Jolla, California, United States of America; Hawaii Pacific University, UNITED STATES

## Abstract

Mitigating direct and indirect interactions between marine predators and fisheries is a motivating factor for ecosystem-based fisheries management (EBFM), especially where predators and fisheries compete for a shared resource. One difficulty in advancing EBFM is parameterizing clear functional responses of predators to indices of prey availability. Alternative characterizations of fishery-predator interactions may therefore benefit the implementation of EBFM. Telemetry data identify foraging areas used by predators and, therefore, represent critical information to mitigate potential competition between predators and fisheries. We analyzed six years (2009–2014) of telemetry data collected at Cape Shirreff, Livingston Island and Admiralty Bay, King George Island, Antarctica, on three species of Pygoscelid penguins and female Antarctic fur seals. In this region, all four species are primarily dependent on Antarctic krill. The tracking data demonstrate local movements near breeding colonies during the austral summer and dispersal from breeding colonies during the winter. We then assessed overlap between predators and the Antarctic krill fishery on a suite of spatiotemporal scales to examine how different data aggregations affect the extent and location of overlap. Concurrent overlap was observed on all spatiotemporal scales considered throughout the Antarctic Peninsula and South Orkney Islands region, including near tagging locations and in distant areas where recent fishing activity has concentrated. Overlap occurred at depths where mean krill densities were relatively high. Our results demonstrate that direct overlap of krill-dependent predators with the krill fishery on small spatiotemporal scales is relatively common throughout the Antarctic Peninsula region. As the krill fishery continues to develop and efforts to implement ecosystem-based management mature, indices of overlap may provide a useful metric for indicating where the risks of fishing are highest. A precautionary approach to allocating krill catches in space would be to avoid large increases in catch where overlap on small spatiotemporal scales is common.

## Introduction

Mitigating direct and indirect interactions between fisheries and marine predators is a motivating factor for ecosystem-based fisheries management (EBFM) worldwide [1]. This is particularly true for the management of fisheries that target low- and mid-trophic level organisms that are major energy pathways from primary production to upper trophic levels [2–4]. In such cases, removal or displacement of potential prey items by a fishery may impact predators by altering, *inter alia*, their foraging behaviors, reproductive success, and survival. However, assigning causation of predator responses to changes in prey availability, including indices such as biomass, recruitment, total catch by a fishery, and harvest rates, remains difficult in many instances [5], but see [6, 7]. Such uncertainty limits the implementation of EBFM.

The recognition of potential competition between predators and fisheries and the desire to minimize the risks that fishing will irreversibly change marine ecosystems, however, lay at the heart of EBFM as implemented by the Commission for the Conservation of Antarctic Marine Living Resources (CCAMLR). In the Atlantic sector of the Southern Ocean, a mid-water trawl fishery for Antarctic krill (*Euphausia superba*) currently catches about 300,000 metric tons of krill in coastal waters of the northern Antarctic Peninsula and island archipelagos of the Scotia Arc [8]. Throughout this region, breeding populations of Adélie (*Pygoscelis adeliae*), gentoo (*P*. *papua*), and chinstrap (*P*. *antarctica*) penguins and Antarctic fur seals (*Arctocephalus gazella*) forage on krill [9–11]. These four species, hereafter “predators”, are studied annually as part of the CCAMLR Ecosystem Monitoring Program (CEMP). The CEMP, implemented in 1985, is a circumpolar research effort designed to detect changes in predator populations and distinguish between changes attributable to fisheries and environmental variation [12]. In particular, the CEMP includes measurements intended to provide information across a range of spatiotemporal scales, from short-term and local-scale impacts (i.e., assessments of foraging effort, diet, chick growth, and reproductive success during the breeding season) to longer-term and larger-scale impacts that integrate over the non-breeding period in habitats remote from local breeding areas (e.g., trends in population size and survival rates) [12].

The predators were chosen because they are central-place foragers (they must forage close to breeding colonies to provision their offspring on regular intervals) whose diets typically contain high proportions of Antarctic krill [9–11]. The restricted foraging space and dietary preference of these predators during the breeding season suggest that fishing activity near breeding colonies may have measurable impacts on the predators. For example, endangered African penguins exhibited a reduction in foraging effort [6] and an increase in chick survival [7] after a no-take marine protected area was established around their breeding colonies, demonstrating that predator performance can be sensitive to the spatial distribution of fishing. Similarly, the predators studied within the CEMP are considered useful indicators for developing fisheries management advice.

Despite the long tenure of the CEMP, however, parameterization of the functional responses of krill-dependent predators to variations in krill biomass and krill catches remains difficult [13–15]. This difficulty likely arises for myriad reasons, including: 1) massive inter-annual variations in krill standing stock due to recruitment [16], advection [17], and changes in krill assessment methodology [18]; 2) strong climate signals in predator reproductive performance and demography [19, 20]; 3) oceanographic and bathymetric conditions that appear to aggregate krill locally [21–23]; and 4) temporary catch quotas intended to minimize local depletion of prey [24]. Additionally, the predators are highly mobile and often disperse widely throughout the Southern Ocean during the austral winter [25–27]. Such dependence on distant foraging habitats may dilute an assessment of local fishing impacts. Nonetheless, krill-dependent predators and the fishery ultimately have a functional overlap; both are dependent on krill. If predators and the fishery use the same population of krill, it follows that removal of krill by one group may limit availability to the other. In the absence of clear functional responses, other metrics of predator-fishery interactions may be useful for informing management decisions.

In systems with resource competition, actors typically minimize competition by partitioning their use of available resources in space and time [28]. Therefore, quantitative descriptions of spatiotemporal overlap between predators and the fishery provide an index of the risk of potential competition for a shared resource. Locations with high overlap might be considered locations where the risks of negative impacts by the fishery are highest. In the Scotia Sea, the extent of concurrent overlap between predator foraging areas and fishing locations is unknown, but potentially widespread throughout coastal regions [29], especially near breeding areas. Bycatches of penguins and seals in krill fishing nets are rare, but have been recorded [30], indicating that overlap can occur at the finest possible scale. We posit, however, that indices of overlap integrated over tens to hundreds of kilometers, and from daily to annual time steps, are relevant to decisions regarding the spatial and temporal distributions of catch.

The krill fishery in United Nations Food and Agriculture Organization Statistical Area 48 is currently managed based on a biomass estimate from an acoustic survey of the Scotia Sea that was conducted in the year 2000 [31]. From this survey, a precautionary catch limit of 5.61 million tons was agreed in 2010 [32]. However, uncertainty regarding the risks to krill-dependent predators that could arise from a spatial concentration of catches has long been the chief concern of fisheries management for Antarctic krill. In 1991, the CCAMLR agreed to an arbitrary interim catch limit (known as the trigger level) of 620,000 tons [33]. This trigger level supersedes the precautionary catch limit until such time that a small-scale spatial allocation of catches above the trigger level can be agreed. Concerned that even catches of 620,000 tons, if concentrated sufficiently, could adversely impact predators, the CCAMLR further agreed to subdivide the trigger level among FAO Statistical Subareas 48.1 through 48.4 [24]. As such, the current catch limit in the Antarctic Peninsula region (Subarea 48.1) is set at an arbitrary 155,000 tons, representing roughly 2.75% of the precautionary catch limit. Thus, current catches are generally regarded as precautionary despite an obsolescing biomass estimate that was distributed primarily in open ocean areas outside the main fishing grounds [34].

Allowing an expansion of the catch up to the precautionary catch limit remains a long-term goal within CCAMLR [35]. Such an expansion will require advice on appropriate spatial allocations of the catch limit and the provision of such advice is an ongoing research focus of the CCAMLR and its working groups [4, 34]. Given the relatively concentrated distribution of krill in the Scotia Sea [36] and a fishery that currently operates exclusively in the Scotia Sea [37], we assume that large increases of krill catch limits imply that existing fishing areas will experience increased fishing pressure. Thus, to identify areas where increased catches may present risk to krill-dependent predators, we assess here the contemporaneous activities of predators and fishing vessels to identify the mosaic of current spatial overlap. We suggest that identifying the areas of concurrent overlap provide information necessary to consider where the risks of increased catches may exist. For example, is risk only present near breeding aggregations or is risk more broadly distributed? We first analyze six years of telemetry data to describe broad patterns of predator movements during summer and winter seasons. We then estimate concurrent overlap of predators with recent fishing activity in time, space, and depth to determine the extent and locations of overlap. We note that these estimates of overlap are not intended to infer negative impacts of current catches on predators. Rather, we identify the multi-species mosaic of overlap with the fishery to identify areas where subsequent increases in catch may increase risks to predator performance.

## Material and Methods

### Ethics statement

All research on seabirds and mammals was permitted and conducted under appropriate Antarctic Conservation Act (Permits 2007–003, 2011–005, 2012–005) Marine Mammal Protection Act (Permits 774-1847-04, 16472–01), and approved animal care and use protocol (seabirds: University of California—San Diego Institutional Animal Care and Use Committee S05480; pinnipeds: National Marine Fisheries Service—Southwest/Pacific Islands Institutional Animal Care and Use Committee 2011–02).

### Study area and data sources

**Fig 1:**
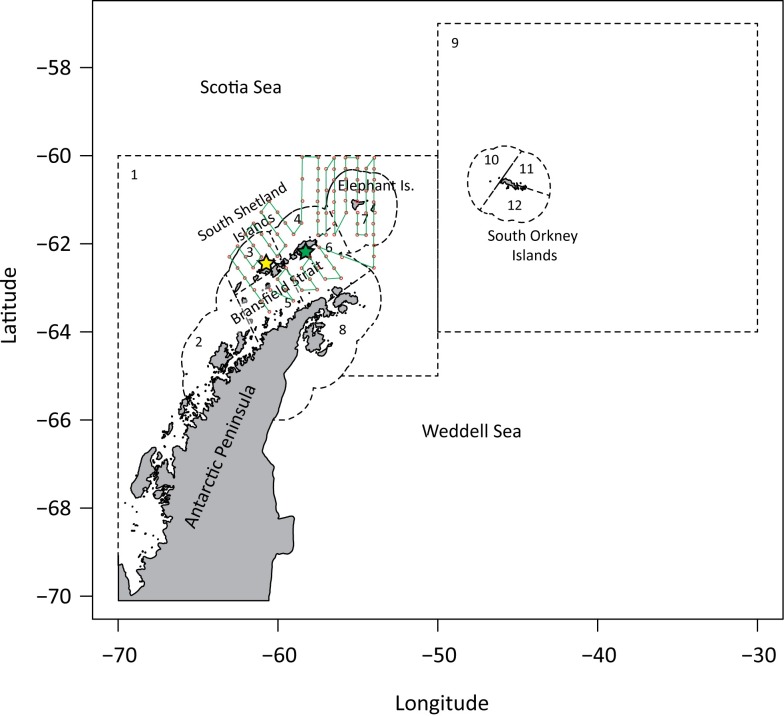
Map of study area, tagging locations (stars), and U.S. AMLR sampling stations (red dots). Acoustic transects occur on the northbound or southbound transits between sampling stations. Boundaries of small-scale management units (SSMUs) are indicated by dashed lines.

At-sea locations of penguins and fur seals were collected with archival and satellite telemetry tags during 2009 to 2014 from long-term research sites at Cape Shirreff, Livingston Island (62.46°S, 60.79°W), and Admiralty Bay, King George Island (62.21°S, 58.42°W). Animals were tracked throughout the south Pacific and south Atlantic sectors of the Southern Ocean, but we focus our analysis of overlap within the United Nations’ Food and Agricultural Organization (FAO) Statistical Subareas 48.1 and 48.2, roughly bounded by 70°W and 30°W longitude, and 70°S and 57°S latitude (Fig 1). Data on krill densities and depth distributions were collected acoustically during scientific research cruises conducted by the U.S. Antarctic Marine Living Resources Program (U.S. AMLR) in the austral summers of 2009–2011 (summer acoustic surveys were not conducted from 2012 to 2014) on an established research grid in waters surrounding the South Shetland Islands (Fig 1). We used haul-by-haul fishing location and net depths recorded by krill fishing vessels that operated in the study area from 2009 to 2014. These fishery data were made available by CCAMLR. The telemetry data, acoustic survey data for krill, and penguin and fur seal abundance data used here are provided in the Supplemental Information (S1 Data).

The period 2009–2014 is representative of a recent shift in fishing operations to more southern areas during winter months that are coincident with the reduced presence of winter sea ice in the Bransfield Strait [38]. During the study period, the sympatric breeding aggregations of Adélie, chinstrap, and gentoo penguins exhibited changes in population size (Table 1) that are mainly commensurate with population trends throughout the Antarctic Peninsula region [39, 40]. One notable exception is the recent positive growth of the Adélie population in Admiralty Bay, though this recent increase must be placed in the context of long-term declines at the site [39]. Antarctic fur seals are only present at Cape Shirreff and represent the southernmost breeding population of this species. Outside of South Georgia, Cape Shirreff is the largest breeding aggregation of fur seals in the Scotia Arc [41, 42]. Pup production at Cape Shirreff, a proxy for population size, declined during the study period (Table 1).

**Table 1:** Instrument deployment and recent population change at each study site. Number of satellite and archival telemetry deployments on penguins and fur seals during the summer and winter (in parentheses) and population size data during the study period, 2009–2014. For the fishery, the number of net tows is reported for each year.

			Annual deployments						Population size		
Tagging location	Species	Instrument	2009	2010	2011	2012	2013	2014	% change	2000	2014
Cape Shirreff	Chinstrap	ARGOS	25	18 (15)	6 (15)	12	10	9	-17.4	4339	3582
		TDR	21	5	3	9	7	6			
	Gentoo	ARGOS	19	18 (14)	10 (14)	12	11	10	-4.6	879	839
		TDR	19	5	3	9	8	6			
	Fur seal	ARGOS	3	2 (20)	-14	0	0	0	-24.2	1569	1188
		GPS	10	7	11	6	5	7			
		TDR	5	5	10	6	5	6			
Admiralty Bay	Adélie	ARGOS	1	4	5	9	10	0	25.9	2577	3246
	Chinstrap	ARGOS	1	3	4	5	4	0	-21.1	361	285
	Gentoo	ARGOS	0	9	13	8	10	5	48.7	4117	6123
Fishery			8691	13289	13532	10262	15513	17591		-	-

### Instrumentation

We used position data from 394 deployments of ARGOS and archival FastLoc^©^ GPS satellite telemetry tags. At-sea positions were estimated during all summer breeding periods, 2009–2014, on penguins with ARGOS tags. Location estimates during the pup-rearing period for fur seals were estimated with both ARGOS (2009–2010) and GPS (2009–2014) tags (Table 1). We also used data collected with ARGOS tags from two overwinter studies of fur seals, chinstrap and gentoo penguins in 2010 and 2011 (Table 1). Summer deployments provided location estimates whenever the tag was wet and for up to one hour after continuous dry conditions were recorded by on-board saltwater sensors. Winter deployments on penguins were duty cycled to provide position estimates for 12 hrs every third day. The ARGOS telemetry instruments used during these tracking studies included SPOT4 and SPOT5 satellite tags (Wildlife Computers, Inc. Redmond, WA, USA), and KiwiSat 101 satellite tags (Sirtrack, Hawkes Bay, New Zealand). The FastLoc^©^ GPS tags were Mk10 tags (Wildlife Computers, Redmond, WA, USA).

Depth data collected during foraging dives by chinstrap and gentoo penguins and fur seals were recorded with time-depth records (TDR) at Cape Shirreff (Table 1). For penguins, foraging dives near breeding colonies were recorded at 1s intervals with Mk9 TDRs (Wildlife Computers, Redmond, WA, USA) from January through February. Fur seal foraging dives over the first 6 foraging trips following parturition, occurring from early December into February, were recorded at 2s intervals using Mk10 TDRs (Wildlife Computers, Redmond, WA, USA). The dive data were processed to identify maximum depths for each dive using Wildlife Computers Instrument Helper (Version 3.0). Only dives exceeding 5m in depth were included in our analysis. The 5m cutoff was used to remove porpoising during travel and is consistent with prior analyses for penguins [43, 44]. The 5m cutoff is deeper than previously used cutoffs for fur seals (e.g. 2m [45]), but inspection of the data suggested that 5m was appropriate, and the estimates of mean maximum dive depths presented here are similar to those previously reported for Antarctic fur seals [45, 46].

All telemetry instruments were attached directly to the back plumage or pelage using adhesives, either quick-drying epoxy or cyanoacrylate glue. For penguins, small plastic cable ties were threaded through underlying feathers and closed over the top of the tag as an additional fastener. Instruments were recovered from all animals that returned to the tagging locations after the summer or winter observation periods. In total, the instrument recovery rate for this study was roughly 75%.

### Krill acoustics

Data on krill densities and depth distributions throughout the U.S. AMLR survey grid (Fig 1) were derived from calibrated acoustic backscatter collected at three frequencies (38, 120, and 200 kHz). The backscatter data collected in the upper 15m of the water column were excluded from analysis due to surface noise. Backscatter data within each 10m depth bin from 15m to 250m and over each 1 nautical mile segment of the survey grid were processed to estimate krill density (g · m^-2^) using the three-frequency stochastic distorted-wave Born approximation (SDWBA) method [16, 47] following standard protocols [18].

### Spatial analysis

We note that raw satellite-based tracking data provide incomplete and often imprecise records of animal movement. We therefore used a state-space modeling approach to derive estimates of overlap with the fishery. Our approach, following the methods of Hinke et al. [27], allows for data-based interpolation of at-sea movements on regular time intervals to improve the characterization of activity at sea. Briefly, we first applied a speed filter [48] to eliminate positions that would require a sustained swimming speed of >2 m·s^-1^ for all species. We next fitted Bayesian state-space models [49] to the data from each deployment and interpolated positions on a 1 hr time step. We then sampled from the posterior distributions surrounding the best fitting tracks to generate 50 alternative track lines for each deployment (again with a temporal resolution of 1 hr). These alternative track lines explicitly account for uncertainty in the location estimates derived from animal-borne instruments [50] and provide distributions of likely positions from which overlap with the fishery can be inferred.

We examined the extent of spatial overlap by binning positions from the 50 alternative tracks representing each deployment on multiple spatial and temporal scales. For spatial scales, we used arbitrary grid sizes of x° longitude by x/2° latitude, where x was 0.25, 0.5, 1, or 2. We also binned positions by small-scale management units (SSMU; Fig 1, Table 2). The SSMUs represent proposed management units for the krill fishery [35]. Each SSMU varies in size (Fig 1, Table 2), but all are larger than our largest spatial grid cell. Likewise, we chose five temporal scales to assess overlap: daily, weekly, monthly, seasonal (two six-month periods from October–March (summer) and April–September (winter)), and annual. Note that the time scales were not used to identify when overlap occurred. Rather, the time scales represented the specific period over which the presence of the fishery and a predator in a particular spatial area would be considered concurrent. These spatial and temporal scales were chosen to range from the scale of a typical net tow by a fishing vessel or foraging trip of a penguin up to annual overlap on proposed fishery management units. For example, a 0.25° longitude by 0.125° latitude grid, equivalent to a 13 km by 14 km polygon at 62°S, is similar to the average maximum distances from tagging locations achieved during summer foraging trips by penguins (19.4 ± 9.75 km) and the average lengths of tows reported by fishing vessels (1.4 ± 0.6 hr, equivalent to 7.8 ± 3.5 km), assuming maximum towing speeds of 3 knots [51].

**Table 2:** Summary of small-scale management units (SSMU) and krill fishing data in the study area, 2009 to 2014. Map number indicates the SSMU number indicated in Fig 1. Data are ordered based on decreasing number of tows conducted in each SSMU.

SSMU	SSMU abbreviation	Map number	Area (x10^3^ km^2^)	N tows	Total catch (tonnes)	Catch per tow (tonnes)
South Orkney West	SOW	10	16.1	23255	379362.7	16.3
Antarctic Peninsula Bransfield Strait West	APBSW	5	22.0	17456	279258.3	16.0
Antarctic Peninsula Bransfield Strait East	APBSE	6	28.7	9283	139733.5	15.1
Antarctic Peninsula Drake Passage West	APDPW	3	15.8	6013	65314.6	10.9
Antarctic Peninsula Drake Passage East	APDPE	4	16.4	3158	34322.0	10.9
Antarctic Peninsula West	APW	2	36.7	3053	43216.1	14.2
Antarctic Peninsula Elephant Island	APEI	7	36.2	818	6680.9	8.2
South Orkney Northeast	SONE	11	10.8	464	7427.1	16.0
South Orkney Pelagic Area	SOPA	9	808.8	303	3493.1	11.5
Antarctic Peninsula East	APE	8	61.6	293	3068.7	10.5
Antarctic Peninsula Pelagic Area	APPA	1	483.4	267	2166.3	8.1
South Orkney Southeast	SOSE	12	15.5	30	414.4	13.8

To estimate overlap for each combination of spatial and temporal scale, we tallied the number of cells where concurrent predator and fishing activities were observed. We present these estimates of overlap in maps that highlight the locations and magnitudes of cumulative presence-presence overlap of instrumented predators and the krill fishery. We also calculated the proportion of cells where concurrent overlap occurred relative to the total number of grid cells occupied by the fishery only, the total number of cells occupied by the predators only, and the total number of cells occupied by both the fishery and the predators. In this way, the overlap results can be interpreted as simple percentages of each respective area used. Finally, we used the Schoener Index [52] to calculate a statistical index of overlap that accounts for both location of overlap and the frequency of concurrent presence of predators and prey in each spatial cell.

### Overlap in depth

We examined overlap in depth distributions by fishery net hauls, mean maximum dive depths made by penguins and fur seals, and the vertical distributions of krill based on acoustic surveys conducted north of Livingston Island. Note that krill density data from acoustic surveys is only available during summers from 2009–2011. All data for this analysis were restricted to the months of January and February and restricted to positions within the boundary of the Antarctic Peninsula Drake Passage West (APDPW) SSMU. This spatial and temporal restriction provided, as close as possible, concurrent estimates of depth use by krill, predators, and the krill fishery in the same local geographic region. We assume that TDR dive locations for penguins occurred within the cluster of locations estimated independently by satellite tracking instruments deployed on different individuals from Cape Shirreff. For fur seals this assumption was not necessary because GPS location and dive data were collected concurrently with the same instrument.

### Statistical reporting

Data summaries are reported as mean ± 1 standard deviation unless otherwise indicated.

## Results

### Temporal extents of fishing and tracking data

During the 6-year study period 7966 vessel-days of fishing were reported in the study area, including 1,380 unique days that accounted for roughly 63% of the study period (Table 3). Raw predator tracking data, collected during 394 deployments, provided 11073 tag-days of data that represented 748 unique days (Table 3). Roughly 80% of the available tracking data was concurrent, on a daily basis, with fishing activity in the study area. The average duration of each deployment on penguins during the breeding season lasted 1 to 2 weeks, while average winter deployments provided data for roughly 3 months (Table 3). The average duration of deployments on fur seals was roughly 3 weeks during summer, while overwinter deployments provided data for more than 5 months (Table 3).

**Table 3:** Summary of satellite telemetry deployments and temporal coverage of fishing activity during 2009–2014. Average deployment duration and maximum distance from tagging locations were calculated from the entire data set. Total and unique days were calculated only for data within the study area (-70S to -57S and -70W to -30W). For the fishery, N is the total number of vessels across all years and the average deployment is the average time, in days, spent in the study area by each vessel.

Location	Period	Species	N	Average (± SD) deployment (d)	Average (± SD) max. distance (km)	Total tag days	N unique days
Cape Shirreff	Summer	Fur seal	51	22.6±13.6	97.4.1±50.5	1194	123
		Chinstrap	79	6.7±4.3	24.3±12.3	613	114
		Gentoo	80	6.8±1.5	15.7±6.1	632	120
	Pre-molt	Chinstrap	1	23.1	259.2	24	24
	Winter	Fur seal	34	167.5±88.0	2186±1343	3481	226
		Chinstrap	30	87.3.6±47.0	1355±1426	1686	203
		Gentoo	28	86.7±56.2	119±82	2452	279
Admiralty Bay	Incubation	Adélie	5	21.8±6.9	294.7±249.2	117	30
	Summer	Adélie	21	8.0±2.1	28.6±19.9	197	57
		Chinstrap	16	8.4±2.5	17.3±11.0	156	53
		Gentoo	45	7.1±2.1	15.3±11.5	389	109
	Pre-molt	Adélie	3	31.7±21.3	646.6±173.5	100	100
		Chinstrap	1	31.4	127.3	32	32
Fishery			66	120.7±79.2	NA	7966	1380

### Predator movements

**Fig 2:**
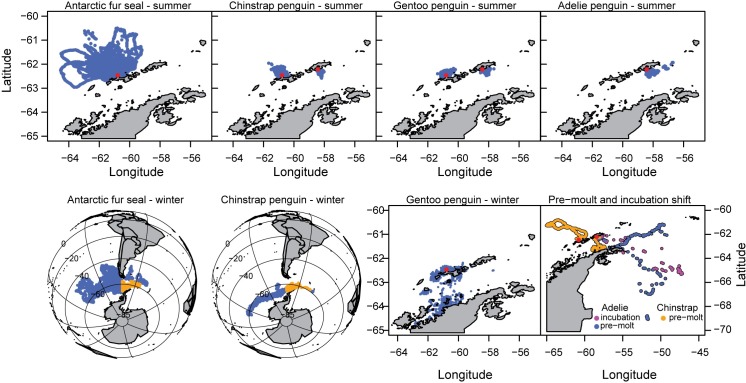
Spatial extent of tracking data from summer and winter deployments for each species. Scales differ for some panels to highlight extent of available data. Overwinter data for fur seals and chinstrap penguins that fall within FAO Subareas 48.1 and 48.2 are highlighted in orange. Incubation shift and pre-molt trips for chinstrap and Adélie penguins are plotted together.

Over the six years of the study, differences in the seasonal movements of each predator species were observed (Fig 2). During the summer breeding season, all predators foraged near their respective breeding locations, with mean maximum foraging ranges being largest for fur seals (97.4 ± 50.5 km) and smallest for gentoo penguins (15.6 ± 6.0 km at Cape Shirreff and 15.3 ± 11.5 km at Admiralty Bay; Table 3). During winter, fur seals and chinstraps exhibited long-distance movements into the South Pacific and South Atlantic. Fur seals radiated from Cape Shirreff to occupy a wide swath of habitats mainly north of 60°S, including remote, pelagic regions of the central South Pacific; waters surrounding southern South America, the Patagonian shelf, and South Georgia. Mean maximum great circle distances from Cape Shirreff were 2187 ± 1343 km and one female fur seal reached a maximum great circle distance of 4750 km from Cape Shirreff. Chinstrap penguins exhibited longitudinal migrations into habitats mainly south of 60°S through the Bellingshausen and Amundsen Seas as far west as 158°W (a great-circle distance of 4782 km from Cape Shirreff). Chinstrap penguins also moved as far east as 26°E (a great-circle distance of 1903 km from Cape Shirreff), near the South Sandwich Islands. Despite these long-distance movements, both fur seals and chinstrap penguins occupied areas throughout the Scotia Sea during winter (Fig 2). Gentoo penguins also moved away from their breeding colonies during winter, but did not disperse as widely as chinstrap penguins or fur seals. In particular, gentoo penguins tagged at Cape Shirreff typically moved into the southwestern Bransfield Strait and occupied coastal areas along the Antarctic Peninsula during winter, with maximum distances from Cape Shirreff averaging 120 ± 82 km. Adélie penguins were not tracked during winter, but incubation shift and pre-molt movements (Fig 2) are indicative of the winter movements undertaken by Adélie penguins that breed in Scotia Arc [24, 25]. The seasonal patterns of movement through the Southern Ocean of all the tracked predators are animated in the Supplemental Information (S1 Animation).

### Overlap in space

**Fig 3:**
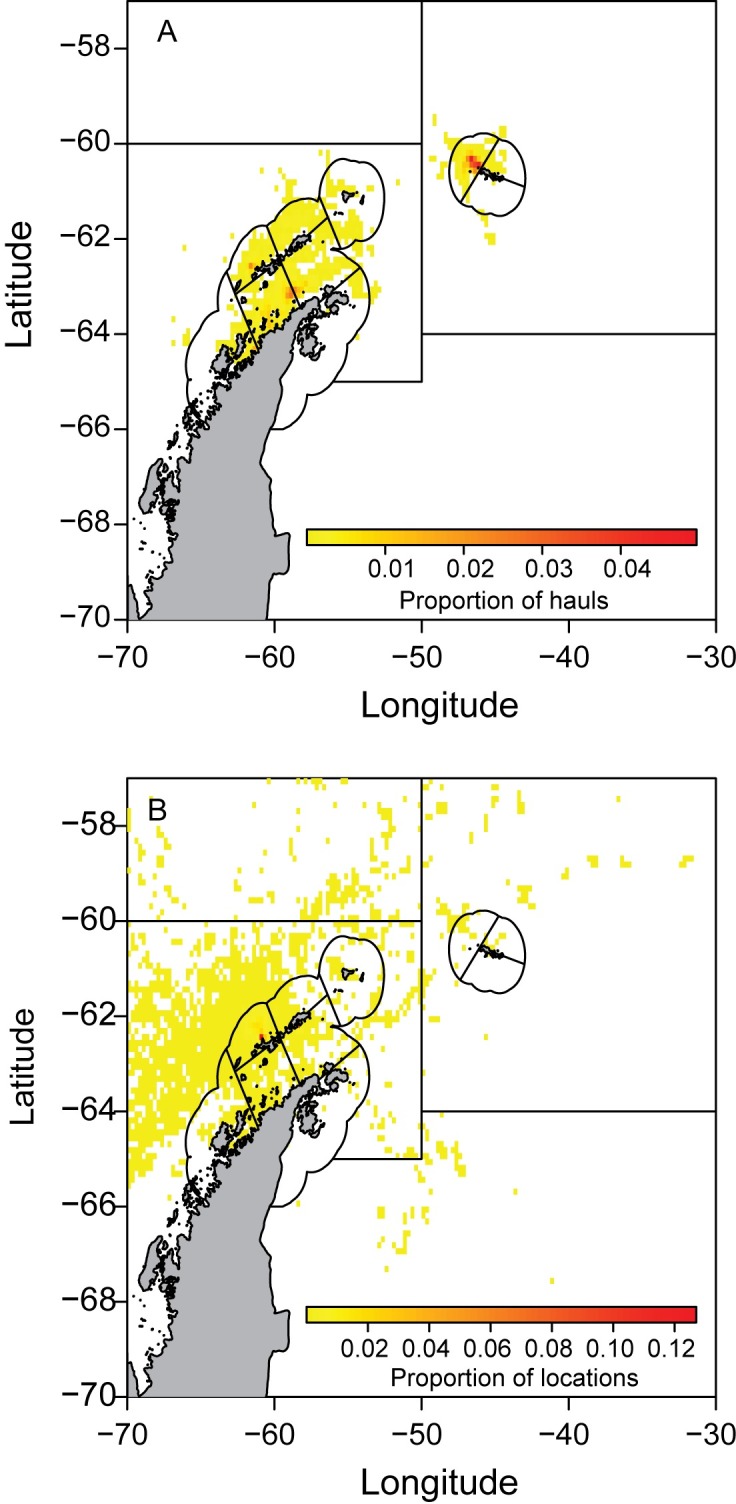
Spatial extent of krill fishing net hauls and raw ARGOS and GPS location estimates. Data are binned to a 0.25° longitude x 0.125° latitude grid. Color scales represent the respective proportion of hauls and location estimates per grid cell.

Fishing was conducted in every SSMU in the study area, but predominantly west of the South Orkney Islands, in the Bransfield Strait, and over the shelf north of the South Shetland Islands (Fig 3A). The numbers of tows and total catches of krill were highest in the South Orkney Islands and within the Bransfield Strait (Table 2). Similarly, raw predator tracking data from the two tagging sites were recorded in all SSMUs except in the SSMU southeast of the South Orkney Islands. In general, the combined spatial distribution of penguins and fur seals tended to be broader than that of the fishery. Despite wide coverage by the predators, we note that the predator location data are concentrated near tagging locations at Cape Shirreff and Admiralty Bay (Fig 3B). An animation depicting seasonal movements of all tracked predators and fishing vessels within Subareas 48.1 and 48.2 is provided in the Supplemental Information (S2 Animation).

**Fig 4:**
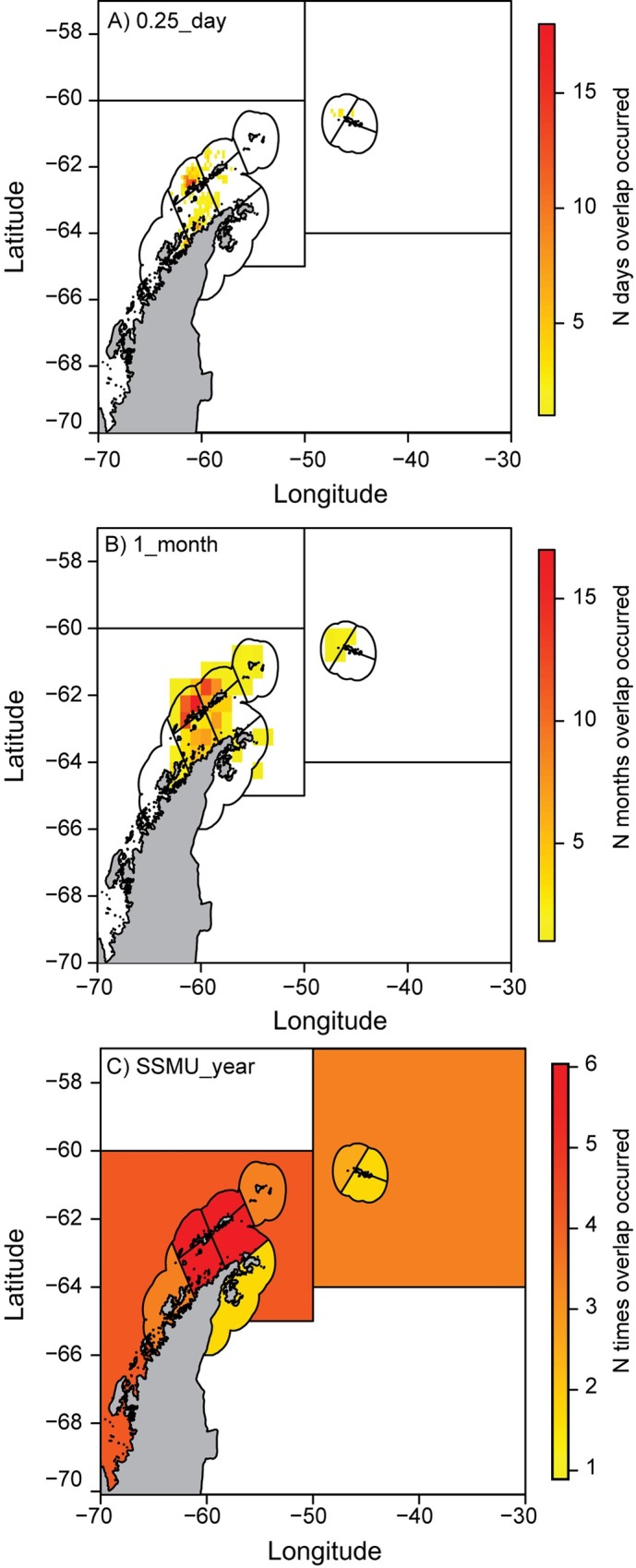
Accumulation of overlap for concurrent predator and fishery presence in a grid cell. A) Spatial grid of 0.25° longitude x 0.125°latitude, temporal scale of 1 day. B) Spatial grid of 1° longitude x 0.5° latitude, temporal scale of 1 month. C) Spatial grid of SSMU, temporal scale of 1 year. Note that scale bars differ for each panel and axes for panel A differ from panels B and C.

There was concurrent spatial overlap of tracked predators and the fishery across the entire range of spatial and temporal scales considered here (Fig 4). Frequent overlap was routinely observed in the vicinity of Cape Shirreff. However, even at the smallest temporal and spatial scales, overlap of tracked predators and the fishery occurred throughout areas north of South Shetland Islands, west of the South Orkney Island groups and within the Bransfield Strait from King George Island in the northeast to the area around Brabant Island and the Danco Coast in the southwest (Fig 4A).

The number of times overlap occurred in any grid cell was typically low. On a daily basis at the smallest spatiotemporal scale, the maximum overlap in any grid cell was 20 days, or roughly 3% of the days with concurrent predator and fishery data (Fig 4A). The frequency of overlap increased with the temporal and spatial scale considered such that maximum overlap occurred in 52% of the months on a 1° longitude x 0.5° latitude grid (Fig 4B) and 100% of the years (Fig 4C) on the SSMU scale.

**Fig 5:**
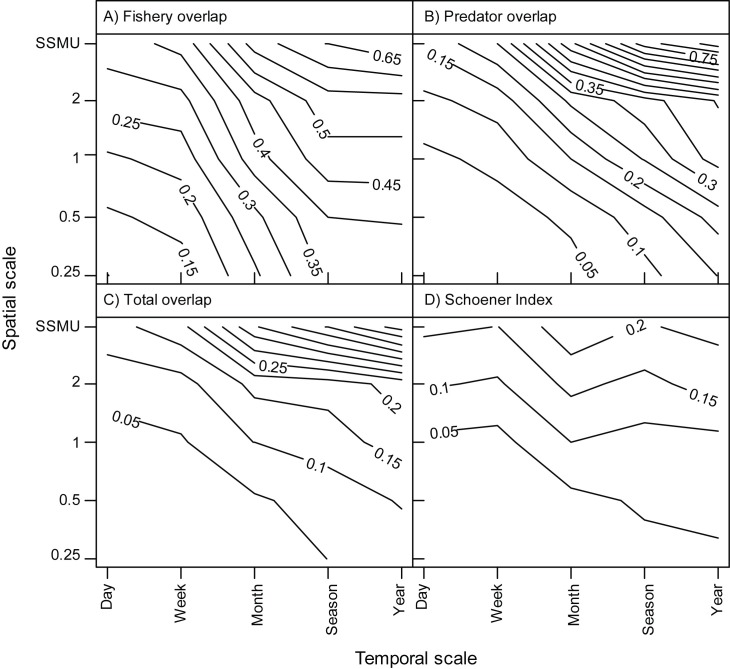
Contours of overlap across space and time. Contours represent the mean proportion of grid cells in which overlap occurs relative to total number of grid cells occupied by the A) fishery B) predators, and C) fishery and predators combined. The Schoener index is plotted in panel D. Spatial scales are identified as 0.25) 0.25° longitude x 0.125°latitude; 0.5) 0.5° longitude x 0.25°latitude; 1) 1.0° longitude x 0.5°latitude; 2) 2.0° longitude x 1.0°latitude.

As the spatiotemporal scale of our analysis increased, the proportion of grid cells where overlap occurred also increased (Fig 5), but the increases occurred at different rates for each respective index of overlap. Overlap with respect to the area occupied by the fishery (Fig 5A) increased most rapidly at the smallest scales, with a mean overlap of 10% on the smallest spatiotemporal scale, 38% at the middle scale (1 month, 1° longitude), and up to 70% of the total fished area at the largest scales. The rapid increase in overlap of the fished area at smaller spatial and temporal scales indicates a relatively constrained distribution of the fishery within the concurrent foraging range of all tracked predators. Relative to the total area occupied by the predators at any one time (Fig 5B), the initial 10% overlap level observed for the fishery area was not achieved until scales larger than weekly and 0.5° longitude were considered and overlap was roughly 15% at the middle scale. Thereafter, overlap increased to near 90% at the largest spatiotemporal scale, indicating that the fished areas overlap almost all of the annual foraging ranges of predators tracked from just two breeding locales. Overlap in the area occupied by both predators and the fishery (Fig 5C) was similar to, but lower than, the pattern of overlap based on predator area alone. The Schoener index, which accounts for both co-occurrence in a grid cell and the proportional use of that area by predators and the fishery, respectively, exhibited a lower absolute level of overlap across all scales, but retained the general pattern of increasing overlap with increasing spatiotemporal scale (Fig 5D).

**Fig 6:**
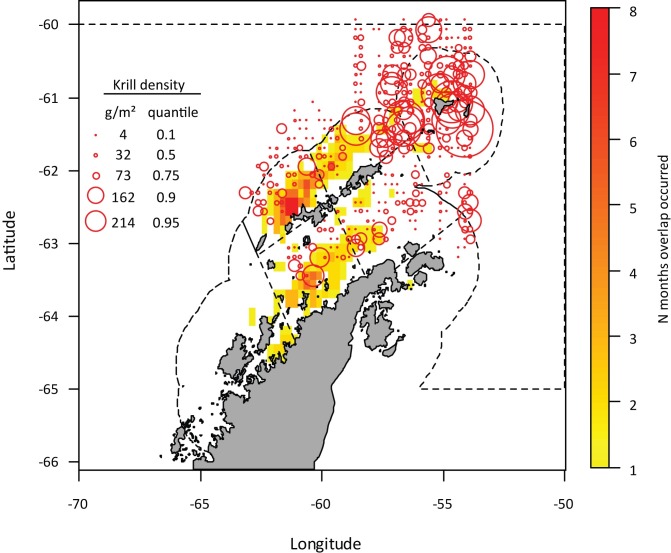
Predator-fishery overlap and mean summer krill density in FAO Subarea 48.1. Data are binned to a 0.25° longitude x 0.125°latitude grid. The time scale for overlap is 1 month. Mean summer acoustic krill density is overlaid as red circles whose diameter is proportional to krill density. The corresponding quantiles of the krill density distribution are indicated in the legend. Boundaries of SSMUs are plotted as dashed lines. Overlap in depth.

Locations where overlap was identified throughout the Antarctic Peninsula region exhibited a mean summer krill density of 58.9 g·m^-2^ (median = 32.8 g·m^-2^; range = 0–385 g·m^-2^; Fig 6), which is similar to the background mean density of krill observed in U.S. AMLR surveys (mean = 60.6 g·m^-2^; median = 31.9 g·m^-2^, range = 0–582 g·m^-2^) across three summers. It is notable that the area around Elephant Island exhibited, on average, the highest estimates of krill density during summer, but very little fishing effort was directed there during the study period (Fig 3A).

**Fig 7:**
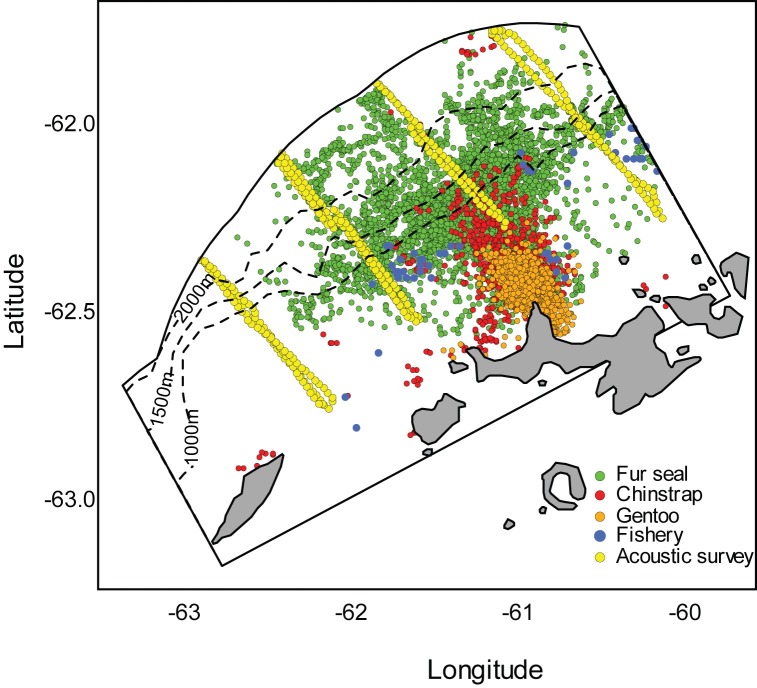
Drake Passage West SSMU acoustic transect, net tow, fur seal, chinstrap, and gentoo penguin locations. All data are from January and February during 2009, 2010, and 2011. Depth contours are included as dashed lines.

**Fig 8:**
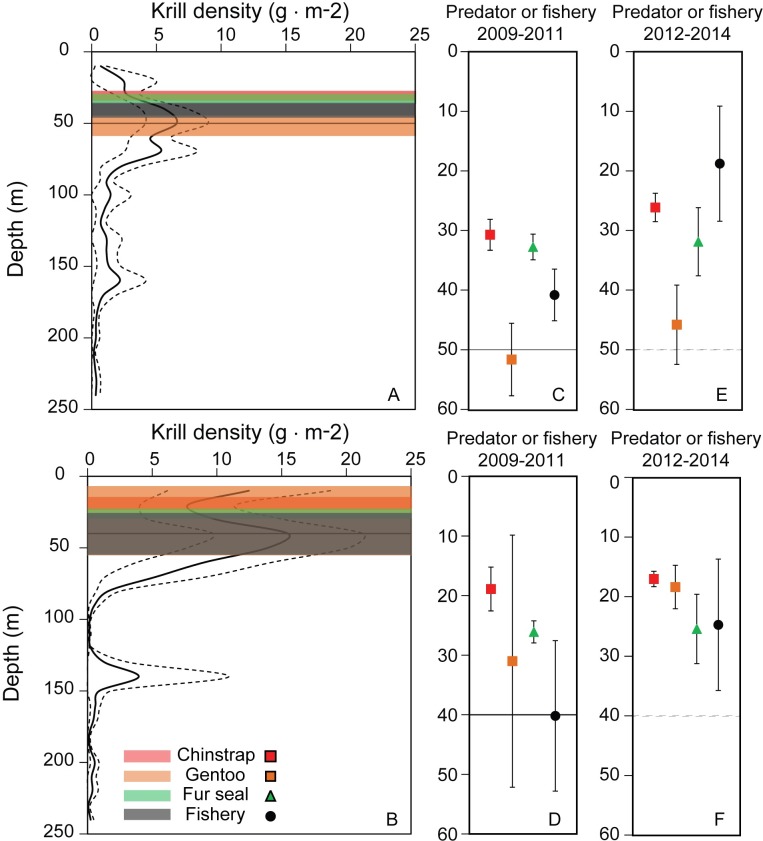
Overlap of krill, predators, and the fishery in depth. A) Overlap during day. B) Overlap during night. Transparent bars in the left hand panels represent mean ± 1 SD in depth use for each predator and the fishery. Average maximum dive depths for predators and average net depth in the fishery for 2009–2012 are plotted at a magnified depth scale for day (C) and night (D) to better illustrate depth ranges; the depth of maximum krill density during day or night is marked with a solid horizontal line in panels A through D. Average maximum dive depth for predators and average net depth in the fishery for 2012 to 2014 are plotted for day time (E) and night time (F); for reference, the depth of maximum krill density from 2009–2012 (no krill density data are available for 2012–2014) is marked as a dashed horizontal line. Note that y-axes in panels C through F differ from panels A and B.

Concurrent data from the krill fishery, acoustic research surveys, and predator tracking recorded during January and February 2009–2011 in the APDPW SSMU (Fig 7) allowed an assessment of overlap in depth at the SSMU scale. Averaged across years, the mean depth distributions of krill exhibited strong diel patterns with significant shoaling and increased krill density in surface waters at night (Fig 8). During daytime, maximum krill density of 6.6 ± 2.4 g·m^-2^ occurred at 50m (Fig 8A), while nighttime maximum densities of 15.5 ± 5.8 g·m^-2^ occurred at 40m. Krill density above 40m was routinely 2x higher during the night than during the day (Fig 8B).

Depth use by predators tracked the diel distributions of krill. During daytime, which accounts for roughly 70% of the time during January and February at Cape Shirreff, the mean maximum depths used by fur seals (32.8 ± 2.2 m) and chinstrap penguins (30.7 ± 2.6 m) were similar, while gentoo penguins generally made deeper dives (51.6 ± 6.1 m; Fig 8C). For all predators, mean maximum dive depths were at or just above the depths where krill density was highest (Fig 8). Fishing depths during daytime averaged 40.9 ± 4.3 and fell between the mean maximum depths of the predators and the depth of maximum krill density. During night time, the mean maximum depths use by fur seals (26 ± 1.8 m), gentoo (31 ± 21.1 m) and chinstrap penguins (18.9 ± 3.7 m) shoaled, and were, on average, 13.0 ± 7.1 m shallower than daytime depths (Fig 8D). Fishing depths at night (40.2 ± 12.6 m) were similar to daytime fishing depths, and matched the depth of maximum krill density at night (Fig 8B). A similar pattern of depth use was observed for all predators from 2012 through 2014 (Fig 8C and 8D). The fishery used shallower net depths during day than either the predators or earlier fishing, but night time depth overlapped all depths used by all predators.

## Discussion

We identified areas and depths with repeated, concurrent overlap of krill-dependent predators and the krill fishery throughout the southern Scotia Arc, particularly in the Bransfield Strait and north of the South Shetland Islands. At the smallest spatiotemporal scales, predator foraging overlapped directly with 12% of the contemporaneous fishing area and fishing overlapped with less than 10% of contemporaneous foraging areas. Though representing relatively small total areas, the small-scale overlaps form a conservative baseline that demonstrates how focal areas of the fishery are used by predators and vice versa. Furthermore, the predators and fishery routinely used similar depth ranges that matched the depths where krill densities were highest during day and night. The evidence for such broad-scale, concurrent overlap in space and depth, despite relatively small numbers of tracked individuals from only two breeding locations, highlights the potential for competitive interactions between predators and the krill fishery at relatively small spatial scales. These results are consistent with modeling work that demonstrates the krill fishery can pose risks to krill-dependent predators [4, 53] and suggest that spatial allocations of catch that prevent concentration of fishing effort in any one area may be warranted.

### Factors affecting overlap

In the Scotia Sea, sea-ice dynamics broadly define the suitability of fishing grounds. Krill-fishing locations change within and between years, driven by changing sea ice conditions that can alter access to preferred fishing grounds [37]. Historically, as winter advanced and sea ice covered southern areas, the fishery moved north and east from the Antarctic Peninsula region toward South Georgia. In recent years, reductions in sea ice extent in the Bransfield Strait during autumn and winter have allowed fishing vessels to remain in southern areas for longer periods of time [37], resulting in the concentration of effort and a large increase in catches taken from Subarea 48.1 relative to earlier decades [38]. Despite the strong dependence on sea ice for access to some fishing areas, however, it is notable that current management may be relatively more restrictive; catches near the 155,000 ton catch limit in Subarea 48.1 have been reached in 4 of the last 6 years [8], prompting closure of the fishery despite conditions that remained favorable to continued fishing operations. As climate change is expected to further reduce the duration of the sea-ice season in the Antarctic Peninsula region [54], longer fishing seasons may be expected.

The extent of spatial overlap we observed was also limited by available tracking data. Our data derive mostly from summer deployments on breeding animals with relatively restricted foraging ranges at two monitoring sites in the South Shetland Islands. Thus, our estimates of overlap with the fishery, while representative of the tracked population, under-represent true overlap given numerous other breeding locations of krill dependent penguins throughout the Scotia Arc [40], untracked demographic groups (e.g., male Antarctic fur seals and juvenile penguins), and the proximity of fishing activity to coastal areas. It is important to note that increasing sample sizes from our study sites may not greatly increase estimates of overlap. Individuals from breeding colonies often exhibit preference for particular, local foraging areas [55] potentially as a means to minimize inter- and intra-specific competition with neighboring populations of central place foragers. While adding more individuals from our tagging sites may increase the frequency of overlap in some grid cells, it is less certain that new areas of overlap would be identified. However, tracking individuals from neighboring colonies throughout the Antarctic Peninsula region or different demographic groups would almost certainly identify more areas of overlap. For example, predator-specific overlap with the fishery differs by species (S1 Appendix), owing to different foraging habitat preferences throughout the year (e.g., Fig 2). However, combining those species-specific overlap maps builds the mosaic of overlap that increases the frequency of overlap in certain places and expands the total area of overlap in general. Additionally, male fur seals are known to occur in the vicinity of the South Shetland Islands throughout the winter [56] and tracking data from geolocators suggests Adélie penguins may forage extensively in areas around the South Orkney Islands during winter [27]. Thus, the spatial extent of overlap observed from our two study colonies, particularly at the smallest spatiotemporal scale, was unexpected.

At the broad scale, the risks to predators in areas of overlap may also be sensitive to movement of krill, whereby variability in the supply of krill from upstream areas dictates the risk of fishing in downstream areas [57, 58]. At finer spatial scales however, heterogeneity in circulation that can arise from interactions between complex bathymetry and time-varying flows may interact with krill behavior (e.g., diel vertical migration), altering the residence time of krill within specific areas relative to the background flux of krill [59, 60]. In such areas, a process of continuous replenishment may misrepresent true dynamics. For example, preliminary analysis of small-scale surface currents from drifter data and circulation modeling (Reiss, unpublished manuscript) suggest that areas with concurrent predator-fishery overlap in the southern Bransfield Strait are characterized by relatively low flows. While large-scale flux is critical for transport of krill from spawning areas in the western Antarctic Peninsula to the eastern Scotia Sea [57, 58], the small scale rates of import and export, and hence any putative benefits of replenishment, in areas of overlap identified here remain unresolved.

### Areas of important overlap

Three general locations of overlap occurred in coastal waters near each tagging location, to the west of the South Orkney Islands, and in the southwestern Bransfield Strait warrant further discussion. The density of data from tagged animals was greatest near the summer breeding colonies of our study animals (Fig 2B). Thus, we expected higher overlap to occur in the vicinity of Cape Shirreff and Admiralty Bay. Indeed, high rates of overlap occurred along the northern shore of Livingston Island and at the mouth of Admiralty Bay (Fig 4A). Fishing activity in the SSMUs adjacent to each tagging site was among the highest in the study region, representing 14.4% of tows and 14.5% of total catch in the Antarctic Peninsula Bransfield Strait East (APBSE) and 9% of all tows and 6.7% of the total catch in the Antarctic Peninsula Drake Passage East (APDPW) during the study period (Table 3). The observed overlap reaffirms prior work to define SSMUs based on likely foraging ranges of penguins and seals [61].

Overlap observed near the South Orkney Islands and in the southwestern Bransfield Strait reveals that potential direct competition for krill between the krill fishery and predators is not limited to local foraging habitats near breeding areas during the summer. Rather, direct predator-fishery interactions may be widespread and occur during winter when predators are distributed more widely. Foraging conditions encountered during winter are considered key drivers of recruitment and overwinter survival in the Antarctic Peninsula region [62]. The extent of concurrent overlap with fisheries in over-wintering habitats provides a mechanism, namely competition, by which predator survival may be further impacted.

The South Orkney West (SOW) and Bransfield Strait West SSMUs experienced the greatest amount of effort by the fishery during the study period (Table 2), accounting for roughly 63% of total net tows and 68% of total catch combined. In both areas, fishing effort occurred throughout the SSMUs, but there were clear concentrations of effort in relatively small areas within each SSMU. The concentration of effort may indicate the presence of predictable aggregations of krill; such aggregations would likely be important foraging areas for predators [23]. With respect to predators in these regions, the confluence of the Scotia and Weddell Seas for Adélie and chinstrap penguins has been documented with tracking data during winter [27] and here we show that concurrent overlap with the fishery also occurred in this general area. These locations near the South Orkney Islands are either focal foraging areas during the non-breeding season (e.g., for Adélie penguins, [27]) or stopovers along migratory corridors (e.g., for chinstrap penguins [25, 27] and fur seals [63]) between the Antarctic Peninsula and the eastern Scotia Sea.

In the Bransfield Strait, there is evidence from acoustic surveys to further support the argument that predictable aggregations of prey are critical for predators. First, overlap in the Bransfield Strait was highest where mean density of krill was greatest (>150g·m^-2^, Fig 6). Furthermore, unpublished data from the U.S. AMLR program suggests that estimates of krill density throughout the Bransfield Strait during winter are higher relative to other locations and seasons around the South Shetland Islands. Predators are known to concentrate foraging effort in such “hotspots” in the Antarctic Peninsula region [64], and the southwestern Bransfield Strait is a key foraging habitat during the non-breeding period for numerous seabirds and marine mammals [64–65]. Given that the background dynamic of sea ice advance and retreat can affect the ability of predators and the fishery to access such hotspots, it is likely that such predictable locations of krill aggregations promote the occurrence of overlap when environmental conditions permit access by the fishery. While the frequency of overlap in these more distant areas was low, the observation of concurrent presence of predators from only two sites and the fishery nonetheless highlights the importance of such small areas within SSMUs for both predators and the fishery.

Finally, the depth distributions of predators and the krill fishery overlapped in the vicinity of Cape Shirreff. Predators tracked the vertical diel migration of krill more closely than the fishery, but the data suggest that all predators and the fishery focused effort on depths where krill density was highest during both day and night. We caution that the comparison of depth use is based on multi-year averages and does not represent concurrent use of vertical habitat. Nevertheless, breeding locations of central place forages may be determined, in part, by proximity to predictable aggregations of prey [21]. If so, then average depth use may provide an integrated estimate of critical foraging habitat. The data suggest that separation between predators during the day was marginally greater (13 ± 10 m) than during night (8.1 ± 3.7 m), consistent with expectations of niche partitioning among predators with similar dietary preferences. In particular, greater krill density throughout the upper 50m of the water column during night may relax competitive conditions among predators and allow greater overlap in depth use. Against this backdrop of depth use by the predators, it is notable that average fishing depths fall directly within the range used by the predators. The shared use of depths with high krill densities by predators and the fishery further support the argument that predators and fisheries compete for krill.

### Implications for EBFM of the krill fishery and beyond

Ecosystem-based fisheries management of fisheries that target forage species, like krill, involves the management of risks to the prey resource, predators, and the marine ecosystem in general [4]. Resolving trade-offs between the acceptable risks to prey, predators, and fisheries is a general challenge for implementation of EBFM. Within the CCAMLR, the concept of the SSMU was an important step toward managing such risks by erecting discrete management units with potentially distinct catch allocations intended to limit the risk of negative impacts of localized fishing on krill-dependent predators. This step may, however, be insufficient; ecosystem modeling work has demonstrated that simply distributing krill catches among SSMUs may not be without risk to dependent predators [4, 53]. Furthermore, our results indicate that direct interactions between predators and fisheries are common at scales smaller than SSMUs. This suggests that allocations of krill catches in the Scotia Sea should consider how to minimize the concentration of fishing effort within SSMUs, particularly if localized fishing overlaps with the foraging ranges of predators. Such spatial limitations on catch are, however, not without risk to performance of the fishery [4, 53].

More generally, while a consensus view is emerging that a relatively large percentage of prey biomass be reserved (i.e. not caught by fisheries) to meet predator demands, e.g., about 33% of standing biomass [66] or 40% of pre-exploitation biomass [2], our results suggest that equal consideration should be given to the spatial distribution of catch limits. Spatial heterogeneity in prey distributions, predator foraging areas, and fishery operations imply that direct interactions of the prey-predator-fishery system often may be localized relative to the larger marine ecosystem being managed. For example, prey density in marine systems may be enhanced by local aggregative mechanisms, whether oceanographic or bathymetric; such areas are likely to be favored by predators and fisheries. This is supported by our maps of overlap, which identified areas where concurrent occupation by predator and fisheries occurred repeatedly over time. A concentration of fishing effort in such localized areas may negatively impact predators, particularly central-place foragers like numerous breeding seabird and pinniped species [2, 3, 67, 68]. To mitigate potential negative effects of fisheries on predators in critical foraging areas, it thus seems necessary to specify not only relatively low harvest rates for forage fish fisheries, but to identify areas where the risk to predators may be greatest and implement management measures that account for high-risk areas. Such information, as presented in simple overlap maps, can advance EBFM efforts by directly including predator-derived indices in a risk-assessment framework.

## Conclusions

Telemetry data provide a clear window into the essential foraging habitats used by marine predators. Identification of these foraging habitats and assessment of their overlap with fisheries that target prey therein can be useful for developing advice on the spatial distributions of catch. Our estimates of overlap identify the northern shelf of the South Shetland Islands and the Bransfield Strait as important areas for both the krill fishery and krill-dependent predators tracked from two field sites in the South Shetland Islands. As the krill fishery continues to develop around the South Shetland Islands and Antarctic Peninsula and the CCAMLR’s effort to implement ecosystem-based management matures, we argue that indices of concurrent overlap estimated at small spatiotemporal scales may provide a useful metric for indicating where the risks of fishing are highest. A precautionary approach to allocating krill catches in space would be to avoid large increases in catch where overlap on small spatiotemporal scales is common.

## Supporting Information

S1 Data Zipped file of telemetry data, acoustic survey data for krill, and penguin and fur seal abundance data used in this analysis. (ZIP)

S1 Animation Animation of the seasonal movements throughout the Southern Ocean of Antarctic fur seals and Adélie, chinstrap, and gentoo penguins, 2009–2014. (MP4)

S2 Animation Animation of the seasonal movements of Antarctic fur seals and Adélie, chinstrap, and gentoo penguins, and the krill fishery in Subareas 48.1 and 48.2, 2009–2014. The small-scale management unit boundaries are drawn for reference.
(MP4)

S1 Appendix Predator-specific maps of overlap with the krill fishery. (DOCX)
