# Supplementary material for: Identifying Risk: Concurrent Overlap of the Antarctic Krill Fishery with Krill-Dependent Predators in the Scotia Sea
Source: PLoS One. 2017 Jan 13;12(1):e0170132. doi: 10.1371/journal.pone.0170132 (PMC5234819; doi:10.1371/journal.pone.0170132)

**S1 Appendix. Predator-specific maps of overlap with the krill fishery**

The aggregate mosaic of overlap presented in the main manuscript (Fig. 4) can be disaggregated to a predator level to address predator-specific overlap with the krill fishery. This allows an assessment of the predator’s respective contributions to the final mosaic of overlap, illustrates how adding new tracking data can alter the inference about where overlap occurs, and highlights how some areas may represent risk to multiple species of predators. Here, we provide such maps at the smallest spatial and temporal scales considered in the manuscript (concurrent overlap on a daily basis in grid cells of 0.25° longitude x 0.125° latitude) to highlight the extent of species-level overlap.

Predator-specific overlap demonstrates differences among species. Notably, overlap with Adélie penguins (represented by the fewest tracking data, see Table 3 in main manuscript) is evident in only one grid cell in the southern Bransfield Strait, while overlap with the remainder of species is more common and extensive. Species-level differences highlight how overlap with gentoo penguins is responsible for the majority of overlap in the Bransfield Strait, while chinstrap penguins and Antarctic fur seals exhibit the most spatially extensive overlap from the South Shetlands through to the South Orkney Islands.

The disaggregation also highlights how the three major regions of overlap discussed in the main text (including the southern Bransfield Strait, the shelf north of the South Shetland Islands, and the area west of the South Orkney Islands arise from habitat use by multiple predators.

**S1 Appendix Fig.1. Predator-specific concurrent overlap with the krill fishery.** Data are binned to a 0.25° longitude x 0.125° latitude grid and overlap is assessed on a daily basis. Note that scale bars differ for each panel.


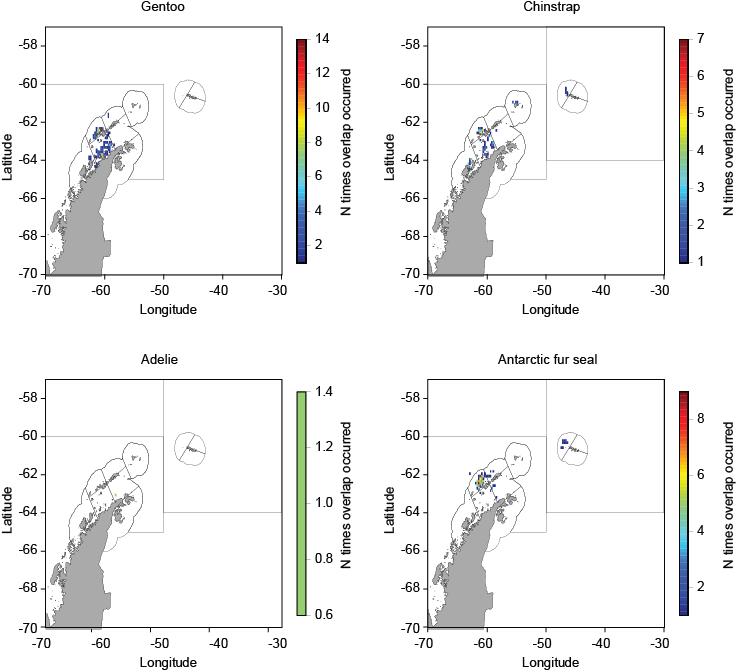

Supplement: S1 Appendix — (DOCX) [file pone.0170132.s004.docx]
